# Lymphome et grossesse: le point de vue de l'obstétricien, à propos de 2 cas

**DOI:** 10.11604/pamj.2014.19.317.4060

**Published:** 2014-11-26

**Authors:** Sarah Amourak, Fatimazahra Fdili Alaoui, Sofia Jayi, Hakima Bouguern, Hikmat Chaara, Moulay Abdelilah Melhouf

**Affiliations:** 1 Service de Gynécologie-Obstétrique 2, Université Sidi Mohammed Ben Abdellah, CHU Hassan II, Fès, Maroc

**Keywords:** LMNH, LMH, grossesse, chimiothérapie, radiothérapie, non-Hodgkin lymphoma, Hodgkin lymphoma, pregnancy, chimiotherapy, radiotherapy

## Abstract

L'association lymphome et grossesse est une situation rare, mais non exceptionnelle, relevant de la coïncidence. Le lymphome vient en 4^ème^ position des cancers rencontrés chez la femme enceinte, La clinique est trompeuse et inconstante, le diagnostic reste histologique et on distingue 2 types: lymphome malin hodgkinien (LMH) et le lymphome malin non hodgkinien (LMNH). Malgré le contexte de véritable urgence hématologique, la décision d'initier ou de différer le traitement est délicate, influencée par le type de lymphome, son extension et sa localisation, la répercussion organique, le degré de maturation f'tale, les risques obstétricaux et les valeurs éthiques, culturelles et religieuses de la patiente. Le traitement consiste essentiellement à une chimiothérapie. Le LH durant la grossesse est relativement moins agressif et d’évolution plus favorable que le LMNH.

## Introduction

L'association lymphome et grossesse est une situation rare, mais non exceptionnelle, relevant de la coïncidence. Le lymphome vient en 4^ème^ position des cancers rencontrés chez la femme enceinte [1]. La clinique est trompeuse et inconstante, le diagnostic reste histologique et le traitement dépend de l’âge gestationnel. Ainsi nous rapportons les particularités diagnostiques, thérapeutiques et pronostiques au cours de la grossesse.

## Patient et observation

A travers ces deux cas colligés au service de gynécologie obstétrique de Fès et une revue de la littérature, nous soulignons les caractéristiques diagnostiques thérapeutiques et pronostiques de cette entité.

## Résultats

### Observation 1

Mme Z N, âgée de 34 ans, suivie depuis un an pour pneumopathie, présentant une toux productive avec quelques épisodes d'hémoptysie, la radiographie du poumon a objectivé une opacité des 2/3 supérieurs de l'hémithorax droit avec de multiples clartés. TDM thoracique a montré un processus lésionnel parenchymo-médiastinal droit avec des adénopathies médiastinales, les 3 BK étaient négatifs. C'est une G3P2, admise pour prise en charge d'une insuffisance respiratoire aigue sur grossesse à terme, en dehors du travail. L’échographie obstétricale a objectivé une grossesse monofœtale évolutive, avec une biométrie à terme, les signes de maturité présents.

Une Césarienne a été indiquée avec extraction d'un nouveau né de sexe F, APGAR 10/10, PN: 3Kg800. Elle a Séjourné en réanimation pendant 3 jours, les suites post opératoires ont été sans particularité, puis elle a bénéficié d'une fibroscopie bronchique avec biopsie d'une infiltration bourgeonnante dont le résultat anatomopathologique est en faveur d'un lymphome non Hodgkinien de phénotype T. Le diagnostic du lymphome lymphoblastique T a été retenu elle bénéficié d'une chimiothérapie.

*L’évolution:* à court terme: la TDM T-A-P a objectivé une nette régression des lésions. Un an après: elle a présenté une altération de l’état général avec orthopnée et toux productive ramenant des expectorations jaunâtres. L'examen clinique objective un syndrome d’épanchement liquidien basithoracique droit, des ‘dèmes des 2 chevilles. La TDM thoracique a montré une volumineuse masse hydrique infiltrant les parties molles thoraciques avec une importante infiltration médiastinale, associé à un épanchement droit.

Sur le plan biologique, elle avait une thrombopénie à 37000 suite à une hypoplasie médullaire post chimiothérapie, confirmée par une biopsie ostéo-médullaire. Puis elle est décédée 17 mois après le début du traitement.

### Observation 2

Mme H.N âgée de 20 ans, primigeste, enceinte de 5 mois, qui a présenté à 3mois de grossesse un syndrome anémique associé à un syndrome hémorragique et une splénomégalie évoluant dans un contexte d'altération de l’état général et de sensation fébrile. La biopsie ostéo-médullaire a montré un LMNH type B d'où la décision d'ITG Elle a été transfusée en per et pré opératoire de 10CP+ 2CG avec uneNFS de contrôle qui a montré Hb: 11g/dl; Plq: 23000.

Le bilan d'extension fait de TDM TAP a montré un épaississement de la paroi supérieure du cavum, hypertrophie des 2 régions amygdaliennes, des adénopathies jugulo carotidiennes, masse médiastinale antérieure et splénomégalie. L’évolution a été marquée un mois après par le décès de la patiente avant de débuter le traitement.

## Discussion

Le lymphome présente un pic de fréquence de 18 à 40 ans, ce qui coïncide avec la période de procréation. L'association lymphome et grossesse a une incidence de 1/6 000 [2]. Certes c'est une grossesse à risque et on distingue 2 types: lymphome malin hodgkinien (LMH) et le lymphome malin non hodgkinien (LMNH).

La symptomatologie clinique est inconstante, il peut se manifester par une fièvre, sueurs nocturnes, vomissement, douleur abdominale, perte de poids, malaise, et parfois par une découverte fortuite de ganglions cervicaux ou sus-claviculaires [3]. Le diagnostic est histologique, il consiste à prélever soit la lésion la plus accessible, soit une zone suspecte profonde pouvant nécessiter une endoscopie ou un geste chirurgical [4].

Le bilan radiologique est indispensable mais il doit être discuté pour que la dose totale d'irradiation délivrée au fœtus soit la plus faible (100mGy). Pour l'imagerie abdomino-pelvienne, l'IRM sans produit de contraste reste l'examen de choix pour une bonne évaluation ganglionnaire et hépatosplénique. L'ultrason abdominal peut aussi être utile. Le PET-CT en revanche est formellement contre-indiqué à tous les stades de la grossesse [5]. Malgré le contexte de véritable urgence hématologique, la conduite thérapeutique est totalement revue. Elle se discute essentiellement en fonction de l′âge gestationnel [5].

### Chimiothérapie et grossesse

Au cours du 1er trimestre il y a un passage de la barrière placentaire, ce qui va entrainer des anomalies morphologiques, des avortements spontanés, tous les types de malformation ont été rapportés, avec une prédominance d′anomalies crânio-faciales et squelettiques. Ces malformations surviennent dans 6 à 24% des cas. Le risque serait plus important en cas de polychimiothérapie [6–8]. Tandis qu'au 2^ème^ et au 3^ème^ trimestre on constate: mort fœtale in utéro, retard de croissance intra utérin (40%), hypotrophie, malformation fœtale et retard mental qui est toujours présent mais faible [6, 9, 10], avec un risque théorique au long court de cancer juvénile, de retard mental et de cytopénie qui a été décrite chez des bébés né jusqu’à 30j après la dernière dose de chimiothérapie surtout chez les prématurés par immaturité du système hépatique et rénale.

Les protocoles de la chimiothérapie diffèrent selon le type du lymphome: Protocol ABVD (adriamycine- bléomycine- vinblastine- dacarbazine) pour le LMH et le protocol CHOP ( adriamycine- cyclophosphamide-vincristine-prédnisone) pour le LMNH.

*Conduite à tenir:* Si une PEC oncologique appropriée peut être initiée, le recours à un avortement thérapeutique peut souvent être évité. Il sera justifié si: Exposition accidentelle aux irradiations ou des agents tératogènes pendant les 10 premières semaines de grossesse; Santé de la mère est mise en danger ( grossesse ou lymphome hautement agressif comme c'est le cas pour notre 2ème patiente); Rechute nécessitant un traitement intensif.

Au cours du 3^ème^ trimestre dès la maturité fœtale, l'extraction s'impose.

Au 2^ème^ trimestre, la décision est difficile; et va être discuté au cas par cas.

### La Radiothérapie

La tératogénicité de la radiothérapie est établie. Les malformations fœtales les plus fréquentes associent microcéphalie, retard mental et troubles oculaires. Un retard de croissance, une hypoplasie génitale, des fentes palatines, et des malformations des oreilles et des orteils sont également observées [5]. La dose totale admissible n′est pas clairement définie elle est donc contre indiqué au cour du 1er trimestre et elle reste une option thérapeutique pour le LMH au cour du 2^ème^ Trimestre.

*L'accouchement* doit être discuté en multidisciplinaire: obstétricien-oncologue-néonatologue, mais la voie basse n'est pas contre indiquée.

*L'allaitement:* Il n’ y a pas de consensus, un cas de neutropénie néonatale a été rapporté chez un bébé nourri au sein durant un traitement maternel par cyclophosphamide [11], il est contre indiqué jusqu’à 2 à 4 semaines après le dernier cycle de la chimiothérapie.

*Pronostic:* il existe des formes graves car elles sont de haut grade histologique, influencé par l'environnement hormonal, l'immunodépression gravidique et le retard diagnostique lié à la grossesse.

Le LH durant la grossesse est relativement moins agressif et d’évolution plus favorable que le LMNH [12].

## Conclusion

La survenue d'un lymphome pendant la grossesse est rare relevant de la coïncidence C'est une grossesse à risque, nécessitant une prise en charge urgente multidisciplinaire. La décision d'initier ou de différer le traitement est délicate, influencée par le type de lymphome, son extension et sa localisation, la répercussion organique, le degré de maturation fœtale, les risques obstétricaux et les valeurs éthiques, culturelles et religieuses de la patiente.
